# Pain Management Strategies for Chronic Pain after Thoracic Surgery: From Pharmacological Therapy to Regenerative Approaches

**DOI:** 10.5761/atcs.ra.26-00089

**Published:** 2026-08-01

**Authors:** I Komang Renteg Sumaarsana, I Gusti Agung Made Adnyana Putra, Ni Made Aprilia Sari

**Affiliations:** 1 Department of Thoracic, Cardiac and Vascular Surgery, Klungkung General Hospital, Klungkung, Bali, Indonesia; 2 Department of Physical Medicine and Rehabilitation, Klungkung General Hospital, Klungkung, Bali, Indonesia

**Keywords:** chronic post-thoracotomy pain syndrome, thoracic surgery procedures, neuropathic pain, pain management, regenerative rehabilitation

## Abstract

**Purpose:**

Thoracic surgery is commonly performed for the management of conditions involving the lungs, mediastinum, pleura, and chest wall. Despite advances in surgical techniques and perioperative care, postoperative pain remains a frequent complication. In some patients, pain may persist and progress into chronic post-thoracotomy pain syndrome (CPTPS), defined as pain lasting more than 2 months after surgery. The prevalence of chronic pain ranges from 21% to 91%, with lower rates observed in minimally invasive approaches such as video-assisted thoracoscopic surgery (VATS) (~35.5%) and robotic-assisted thoracic surgery (RATS) (11.2%–34.6%).

**Methods:**

This study presents a literature review evaluating the epidemiology, pathophysiology, and management of CPTPS, including pharmacological, interventional, and rehabilitation-based approaches, as well as emerging biological therapies.

**Results:**

Chronic pain is multifactorial, involving tissue injury, inflammation, intercostal nerve damage, and central sensitization. It is associated with impaired respiratory function, reduced mobility, sleep disturbances, and decreased quality of life. Multimodal analgesia and regional anesthesia are key strategies, while rehabilitation supports functional recovery. Emerging biological therapies show promise but remain limited in evidence.

**Conclusion:**

A multidisciplinary approach is essential to reduce CPTPS and improve long-term outcomes and quality of life.

## Introduction

Thoracic surgery is a commonly performed intervention in the management of various conditions affecting the thorax, particularly those involving the lungs, mediastinum, pleura, esophagus, and chest wall.^1)^ Despite advancements in surgical techniques and perioperative care that have improved patient prognosis, postoperative pain remains a common complication after thoracic surgery. This is related to the involvement of complex chest wall structures such as the ribs, intercostal muscles, and intercostal nerves, which are often exposed to intraoperative manipulation and trauma.^2)^ As a result, thoracic surgical procedures are often associated with greater postoperative pain intensity than other surgical interventions.^3)^

In most patients, postoperative pain is temporary and improves with tissue healing. However, in a subset of patients, pain may persist and progress into chronic post-thoracotomy pain syndrome (CPTPS), defined as persistent or recurrent pain lasting more than 2 months after thoracic surgery without other identifiable causes, such as infection or underlying disease.^3,4)^ Several studies have reported that the prevalence of CPTPS ranges from 21% to 91%, with lower rates observed in patients undergoing video-assisted thoracoscopic surgery (VATS), at approximately 35.5%. These variations may be influenced by several factors, including the type of surgical procedure, duration of follow-up, and methods used for pain assessment.^5–7)^

The development of CPTPS is multifactorial, involving tissue injury during the surgical procedure, inflammatory responses, and intercostal nerve damage resulting from tissue manipulation. Injury to the intercostal nerves may lead to neuropathic pain, characterized by burning, stabbing sensations, or hypersensitivity in the chest wall. In addition, the central nervous system plays a role in maintaining pain perception even after tissue healing has occurred.^3,8)^ This condition has significant clinical implications, including impaired respiratory function and limitations in physical activity, which adversely affect patients’ quality of life. It may also reduce patient satisfaction with surgical outcomes, underscoring the importance of effective pain management in postoperative thoracic care.^2,6)^

A range of strategies has been developed to manage postoperative pain following thoracic surgery, including pharmacological treatment, regional analgesia such as nerve blocks, and rehabilitation approaches to improve respiratory function and mobility. Multimodal analgesia not only alleviates pain but also helps prevent the transition from acute to chronic pain.^2,9)^ In recent years, more innovative therapeutic approaches, such as cell-free therapy, cell-based therapy, and the concept of regenerative rehabilitation, have gained increasing attention as potential strategies for modulating inflammation, promoting neural repair, and optimizing tissue healing.^10,11)^ Therefore, this literature review aims to evaluate the prevalence, pathophysiology, and various pain management strategies for CPTPS, with a particular focus on improving postoperative quality of life and patient satisfaction.

## Definition and Clinical Scope of Chronic Pain after Thoracic Surgery

Chronic postoperative pain refers to persistent or recurrent pain that develops after a surgical procedure and persists beyond the expected period of normal tissue healing, typically lasting more than 2 to 3 months postoperatively. In the context of thoracic surgery, this condition is referred to as CPTPS, characterized by persistent pain at the incision site or chest wall in the absence of other identifiable causes, such as infection, disease recurrence, or postoperative complications.^3,4)^ From a clinical perspective, CPTPS exhibits heterogeneous features and may involve nociceptive, neuropathic, or mixed pain components.^12)^ Neuropathic pain frequently predominates as a result of intercostal nerve involvement during surgery, manifesting as characteristic symptoms such as burning or stabbing sensations, paresthesia, and hypersensitivity to light stimuli.^6)^ In addition, patients may report pain triggered by mild activities such as coughing, deep breathing, or body movement, which directly impacts respiratory function and daily activities.^3)^

The clinical scope of CPTPS extends beyond pain perception alone and also includes associated functional and psychological impacts. Persistent pain may lead to reduced mobility, impaired pulmonary ventilation due to limited deep breathing, and decreased sleep quality.^3,6)^ In the long term, this condition may contribute to reduced quality of life and increased anxiety and depression, as well as dependence on analgesic use.^4,13)^ Therefore, a comprehensive understanding of the definition and clinical spectrum of CPTPS is essential as a foundation for determining appropriate and effective management strategies.

## Epidemiology and Prevalence

The incidence of CPTPS varies widely across studies, reflecting both the complexity of the condition and heterogeneity in study design, pain definitions, and follow-up duration. Reported prevalence rates range from 21% to 91% in patients undergoing thoracic surgery, with higher estimates observed in studies employing specific pain assessment methods.^5,6)^ In minimally invasive approaches such as VATS and robotic-assisted thoracic surgery (RATS), the prevalence of chronic pain has been reported to be lower, at approximately 35.5% for VATS and ranging from 11.2% to 34.6% for RATS. Although both techniques demonstrate lower rates compared with open thoracotomy, no significant difference in the incidence of chronic pain has been observed between VATS and RATS.^7,14)^ A summary of reported prevalence rates of CPTPS across various surgical techniques is presented in **Table 1**.

**Table 1:** Epidemiology and prevalence of chronic pain after thoracic surgery

Study	Year	Technique	Prevalence	Follow-up	Assessment
Kim et al.	2021	Thoracotomy	21%–91%	Variable	Questionnaire
Chen et al.	2023	VATS	~35.5%	≥3 months	Meta-analysis
Tokuishi et al.	2024	RATS	11.2%–34.6%	≥3–6 months	Clinical

VATS, video-assisted thoracoscopic surgery; RATS, robotic-assisted thoracic surgery

A key factor contributing to the variability in prevalence is the surgical technique employed. Open thoracotomy is consistently associated with a higher incidence of chronic pain compared with minimally invasive approaches, including VATS and RATS.^3)^ This is related to the greater degree of tissue trauma involved, including the use of rib retractors and a higher risk of intercostal nerve injury associated with open thoracotomy. Nevertheless, it is important to note that chronic pain may still occur in patients undergoing VATS or RATS; therefore, these approaches do not completely eliminate the risk of developing CPTPS.^14)^

In addition to surgical technique, variations in prevalence are also influenced by methodological factors. Differences in the definition of chronic pain, particularly regarding its duration (e.g., more than 2 months versus 3 months), can affect the reported prevalence rates. The length of the follow-up period also plays an important role, as prevalence may decrease over time. Furthermore, variations in pain assessment methods such as Numeric Rating Scales (NRSs), neuropathic pain-specific questionnaires, and quality-of-life evaluations contribute to heterogeneity across study findings.^6,15)^ Variations in the type of surgical procedures, including lung resection, mediastinal surgery, and chest wall procedures, may also influence the incidence of chronic pain.^3)^

Overall, the high prevalence and variability of CPTPS highlight that chronic pain after thoracic surgery is a significant clinical problem and remains a challenge in modern surgical practice. Therefore, a comprehensive understanding of its epidemiology is essential as a foundation for developing more effective prevention and management strategies.^3)^

## Surgical Techniques and Their Relationship to Chronic Pain

Surgical technique is a key determinant of the risk of CPTPS. Differences among procedures, including open thoracotomy, VATS, sternotomy, and interventions involving rib retraction or chest tube placement, lead to varying degrees of tissue injury, which directly affect the development of CPTPS. More invasive approaches, particularly open thoracotomy, are associated with higher rates of chronic pain, largely due to greater mechanical trauma to tissue and neurovascular structures.^2,7,16)^ In contrast, minimally invasive techniques such as VATS and RATS are generally associated with lower levels of pain due to reduced tissue injury, although the risk of chronic pain cannot be completely eliminated.^3)^

The primary mechanism underlying the relationship between surgical technique and chronic pain is related to the degree of trauma to the thoracic wall and the involvement of intercostal nerve structures. In open thoracotomy, the use of rib retractors results in significant rib spreading, thereby increasing the risk of compression or direct injury to the intercostal nerves.^2,13)^ In addition, muscle division, pleural irritation during intraoperative manipulation, and subsequent scar formation during the healing process all contribute to the persistence of postoperative pain.^3,8)^ Intercostal nerve injury plays a crucial role in the development of neuropathic pain, which constitutes a dominant component in many cases of CPTPS. To minimize this risk, various modifications in surgical techniques have been developed, such as muscle-sparing and nerve-sparing approaches, which aim to reduce tissue damage and preserve the integrity of the intercostal nerves during surgery.^2,4)^

In contrast, minimally invasive approaches such as VATS and RATS are designed to minimize tissue trauma through smaller incisions and without the need for extensive rib spreading.^6)^ This contributes to reduced acute pain intensity and facilitates faster postoperative recovery. However, several pain mechanisms may still occur, including pleural irritation, localized trauma from port placement, and intercostal nerve injury due to instrument pressure or positioning. In addition, other factors, such as chest tube placement, can serve as significant sources of pain in both the acute and chronic phases.^3,7,16)^

Comparative studies between open thoracotomy and VATS indicate that, despite the benefits of minimally invasive approaches in reducing postoperative pain, the risk of chronic pain remains and cannot be entirely eliminated.^3)^ These findings indicate that the development of CPTPS is not solely influenced by surgical technique but also by other factors such as inflammatory response, patient characteristics, and the quality of perioperative pain management. Therefore, appropriate selection of surgical techniques should be accompanied by strategies to minimize nerve injury and implement comprehensive pain management to reduce the risk of chronic postoperative pain.^2,8)^

Overall, surgical technique not only determines the success of the operative procedure but also has significant implications for long-term pain outcomes. A comprehensive understanding of the relationship between surgical techniques and the mechanisms underlying pain development, including the application of muscle-sparing and nerve-sparing principles, is essential for developing more optimal strategies. Such an approach may help to reduce the incidence of CPTPS and improve patients’ quality of life following thoracic surgery.^2–4)^

## Selection of Analgesic Strategies According to Surgical Approach

The choice of perioperative analgesic strategy in thoracic surgery should be individualized according to the surgical approach, extent of tissue injury, expected postoperative pain intensity, and patient-related factors.^2,9)^ Differences between open thoracotomy and minimally invasive procedures, such as VATS and RATS, influence both the severity of postoperative pain and the optimal analgesic modality.^3,7,14)^

Open thoracotomy is generally associated with greater tissue trauma, extensive rib spreading, and a higher risk of intercostal nerve injury, resulting in more severe postoperative pain and an increased risk of CPTPS.^2,3,13)^ Therefore, thoracic epidural analgesia (TEA) has traditionally been considered the gold standard for pain control in open thoracotomy because of its effectiveness in providing extensive segmental analgesia and improving respiratory function. Nevertheless, TEA may be associated with adverse effects, including hypotension, urinary retention, and rare neurological complications, which require careful patient selection and monitoring.^2,9)^

In minimally invasive procedures such as VATS and RATS, postoperative pain is generally less severe because of reduced tissue trauma and the absence of extensive rib spreading.^3,7,14)^ Consequently, less invasive regional analgesic techniques, including paravertebral block (PVB), erector spinae plane block (ESPB), serratus anterior plane block (SAPB), and intercostal nerve block (ICNB), have gained increasing popularity. These techniques may provide effective analgesia with fewer systemic adverse effects and may facilitate enhanced recovery and early mobilization.^2,9,16)^

ICNB is commonly performed during minimally invasive thoracic surgery, particularly through direct infiltration under surgical visualization. This technique is technically simple and may reduce opioid requirements during the early postoperative period.^2,9)^ However, the duration of analgesia is relatively limited compared with TEA or PVB, and repeated administration or continuous catheter techniques may be required in selected patients.^9)^

In addition to the choice of regional anesthesia technique, multimodal analgesia remains essential across all surgical approaches.^2,12)^ The combination of regional anesthesia, non-opioid analgesics, opioid-sparing strategies, and rehabilitation interventions may reduce acute pain severity and potentially decrease the transition from acute postoperative pain to CPTPS.^2,8)^ Therefore, analgesic strategy selection should not only consider surgical invasiveness but also patient-specific risk factors, perioperative goals, and postoperative recovery pathways.^13,16)^

## Risk Factors for Chronic Pain after Thoracic Surgery

CPTPS is a multifactorial condition arising from complex interactions between patient-related, surgical, and postoperative factors. A comprehensive understanding of these risk factors is crucial for identifying high-risk patients and guiding the development of more effective preventive strategies.^8,17)^

### Patient-related factors

Patient-related characteristics are important determinants of the risk of chronic postoperative pain. Evidence suggests that younger patients are more likely to develop chronic pain, potentially due to heightened pain sensitivity and more robust inflammatory responses. Furthermore, female sex has been associated with an increased risk, possibly reflecting hormonal influences and differences in pain perception.^13,17)^

Psychological factors represent another important determinant in the development of chronic postoperative pain. Conditions such as anxiety, depression, sleep disturbances, and pain catastrophizing are known to increase pain perception and facilitate the transition from acute to chronic pain. In addition, patients with a prior history of chronic pain or heightened pain sensitivity are at greater risk of developing CPTPS. Therefore, assessment of psychological status and preoperative pain history is an essential component of a comprehensive perioperative approach.^8,13,18)^

### Surgical factors

Surgical technique and procedural characteristics play a significant role in the development of chronic postoperative pain. Open thoracotomy has consistently been associated with a higher risk than minimally invasive approaches, largely attributable to the greater degree of tissue trauma involved.^3)^ In addition, longer operative duration has been associated with an increased risk of chronic pain, likely due to prolonged exposure to tissue injury and inflammatory responses.^8,13,12)^

Furthermore, intercostal nerve injury represents a key component in the pathogenesis of CPTPS. In more invasive procedures, the use of rib retractors resulting in significant rib spreading, extensive tissue dissection, and substantial intraoperative manipulation can increase the risk of intercostal nerve compression or injury. This contributes to the development of neuropathic pain, which often constitutes a dominant component in CPTPS.^2,4,13)^

### Postoperative factors

Postoperative factors also play a crucial role in determining whether acute pain progresses to chronic pain. Inadequately controlled acute pain during the early postoperative period is one of the strongest predictors of chronic pain, as it is associated with the development of peripheral and central sensitization, which amplify pain transmission.^4,8)^ In addition, postoperative complications such as infection, atelectasis, and respiratory dysfunction may exacerbate pain intensity and prolong the recovery process.^6)^ The duration of chest tube placement may also serve as a source of ongoing irritation to the pleura and intercostal nerves, thereby contributing to the development of persistent pain.^7,17)^ In addition, delayed mobilization and postoperative rehabilitation may further impair respiratory function and increase pain perception, thereby increasing the overall risk of CPTPS.^16)^

### Clinical implication

Overall, these factors demonstrate that chronic pain following thoracic surgery is not solely determined by surgical technique, but rather results from a multidimensional interaction of biological, psychological, and clinical factors.^8)^ Therefore, a comprehensive approach is required, encompassing optimization of surgical techniques, adequate perioperative pain management, and assessment and management of patient-related psychological factors. This integrated approach is expected to reduce the incidence of CPTPS and improve long-term patient outcomes.^3,16)^ A comprehensive overview of the major risk factors, including patient-related, surgical, and postoperative contributors, is presented in **Table 2**.

**Table 2:** Risk factors for chronic post-thoracic surgical pain

Category	Factor	Mechanism	Implication
Patient	Younger age	High sensitivity	Higher risk
Patient	Female sex	Hormonal	Higher incidence
Psychological	Anxiety/depression	Central sensitization	Persistence
Surgical	Thoracotomy	Nerve injury	High risk
Postop	Severe acute pain	Sensitization	Chronic pain

## Pathophysiology of Chronic Pain after Thoracic Surgery

**Fig. 1:**
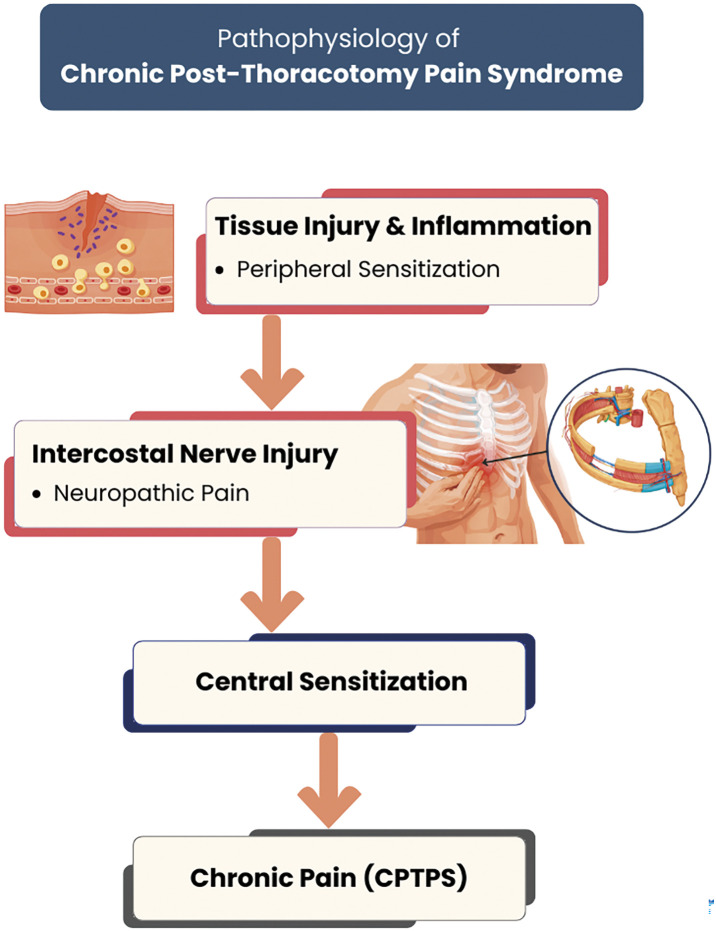
Pathophysiological mechanisms of CPTPS, including peripheral sensitization, intercostal nerve injury, and central sensitization leading to persistent pain. CPTPS, chronic post-thoracotomy pain syndrome

CPTPS arises from complex interactions between peripheral and central mechanisms initiated during the early phase of tissue injury. These processes include activation of nociceptive pathways, nerve damage, and neuroplastic changes in the central nervous system, which progressively contribute to persistent pain even after tissue healing has occurred.^8)^ Therefore, a comprehensive understanding of this pathophysiology is essential as a foundation for developing more effective prevention and management strategies.^3)^ The underlying mechanisms of CPTPS are illustrated in **Fig. 1**.

### Peripheral mechanisms

In the early postoperative phase following thoracic surgery, tissue injury resulting from incision, dissection, and intraoperative manipulation triggers a local inflammatory response. This process is characterized by the release of multiple inflammatory mediators, including prostaglandins, bradykinin, proinflammatory cytokines, and substance P, which collectively enhance nociceptor sensitivity. This activation and sensitization lead to a lowered pain threshold, whereby previously non-painful stimuli may be perceived as painful or elicit exaggerated pain responses.^8,19)^ In addition, persistent inflammation may sustain activation of nociceptive pathways, thereby prolonging the acute pain phase and increasing the risk of pain chronification. Therefore, effective control of inflammation and pain during the early phase is essential to prevent the development of chronic pain.^3,8)^

### Neuropathic mechanisms

In addition to nociceptive mechanisms, intercostal nerve injury during surgical procedures plays a significant role in the development of chronic postoperative pain with a neuropathic component. Such injury may occur due to compression, stretching, or transection of the nerve during rib retraction or tissue manipulation.^6,8)^ Nerve damage may result in neuroma formation, ectopic nerve activity, and alterations in neural signal transmission. Clinically, this manifests as characteristic neuropathic symptoms such as burning, stabbing, tingling sensations, and dysesthesia over the chest wall. This neuropathic component frequently predominates in CPTPS and contributes to the complexity of its management.^6,8,20)^

### Central sensitization

The persistence of pain after thoracic surgery also involves changes in the central nervous system through central sensitization. Sustained peripheral nociceptive input promotes increased excitability within spinal neurons, leading to amplification of pain signaling and altered central processing. This process is accompanied by various neuroplastic changes, including increased activity of excitatory neurotransmitters and reduced inhibitory pain modulation.^8,19)^ As a result, the central nervous system becomes more responsive to various stimuli, including those that were previously non-painful. Central sensitization also explains why pain may persist despite the healing of the initial tissue injury and represents a key mechanism in the transition from acute to chronic pain.^13,19)^

### Transition from acute to chronic pain

The transition from acute to chronic pain is a dynamic process influenced by multiple biological and clinical factors. Severe and inadequately controlled acute pain in the early postoperative period has been identified as one of the strongest predictors of chronic pain development.^4,8)^ This association may be explained by the ability of high pain intensity to potentiate nociceptive pathway activation and induce both peripheral and central sensitization.^19,20)^ In addition, prolonged inflammatory responses, nerve injury, and psychological factors also contribute to the process of pain chronification. Therefore, efforts to prevent chronic pain should begin in the perioperative phase through a multimodal approach, including adequate pain control, the application of surgical techniques that minimize nerve injury, and optimization of patient condition both before and after surgery.^8,16)^

## Clinical Impact of Chronic Pain after Thoracic Surgery

CPTPS is not merely a subjective complaint but has broad clinical implications for physiological function, psychological well-being, and overall quality of life. These effects may persist in the long term and significantly influence postoperative outcomes.^6,13)^

A key clinical impact is the impairment of ventilation and respiratory function. Chest wall pain can limit deep inspiration and suppress the cough reflex, resulting in reduced lung expansion. Consequently, this may increase the risk of atelectasis, secretion retention, and pulmonary infection.^21)^ These findings are consistent with the study by Schieren et al., which reported that postoperative complications following thoracic surgery occur in approximately 27.7% of patients, with respiratory complications being the most common and having the greatest impact on mortality and length of hospital stay.^22)^ In the long term, impaired ventilation may delay the recovery of respiratory function and further compromise patients’ functional capacity.^16,22)^

Persistent pain frequently results in movement avoidance, especially during activities that require chest wall expansion, such as ambulation, lifting, or respiratory exercises. This can lead to reduced mobility, physical deconditioning, and delayed rehabilitation, ultimately slowing functional recovery and increasing the risk of complications.^4)^ Chronic pain not only causes physical discomfort but also contributes to sleep disturbances. Persistent pain or pain that worsens in certain positions can make it difficult for patients to initiate and maintain sleep.^13,23)^ These sleep disturbances may further exacerbate pain perception, creating a self-reinforcing cycle between pain and impaired sleep.^24)^

Cumulatively, persistent chronic pain, together with impaired respiratory function and limitations in physical activity, contributes to a decline in patients’ quality of life. This condition may lead to difficulties in performing daily activities, reduced productivity, and limitations in social interactions.^3,6)^ A recent study by Yaman et al. demonstrated that CPTPS not only has a high prevalence but also significantly interferes with daily activities and patient function, with more than half of patients reporting activity limitations due to persistent pain.^4)^ In addition, postoperative complications in thoracic surgery, which remain relatively common, may contribute to worsening clinical outcomes, including increased mortality and prolonged hospital stay, thereby indirectly impairing patients’ quality of life.^22)^

CPTPS affects not only physical health but is also closely associated with increased anxiety, depression, and emotional distress. These conditions may exacerbate pain perception through psychoneuroimmunological mechanisms, including central sensitization, thereby creating a self-reinforcing cycle between pain and psychological distress. Furthermore, psychological disturbances may negatively impact patients’ adherence to treatment and rehabilitation programs.^8,19)^ These findings are consistent with the study by Chen et al., which demonstrated that psychological factors such as anxiety and depression are important predictors of the development and persistence of chronic postoperative pain.^25)^ Furthermore, psychological factors, including pain catastrophizing and emotional stress, can amplify pain perception and perpetuate the cycle of chronic pain.^18)^

Although the primary goal of thoracic surgery is to treat the underlying disease, persistent chronic pain may reduce patient satisfaction with surgical outcomes. Patients may perceive that the benefits of surgery do not outweigh the discomfort experienced, particularly when pain interferes with daily activities. A study by Yaman et al. demonstrated that CPTPS is associated with activity limitations and reduced quality of life, ultimately influencing patients’ perceptions of surgical success.^4)^ In addition, chronic pain has been shown to contribute to patient dissatisfaction with surgical outcomes and long-term results.^6,12)^

Chronic pain may also lead to long-term use of analgesics, including medications with significant potential side effects. Dependence on analgesics, particularly opioids, can result in additional complications such as tolerance, systemic adverse effects, and the risk of misuse. Studies have shown that patients with chronic postoperative pain often require prolonged analgesic therapy, which increases the risk of sustained opioid use and associated adverse effects.^8,12)^ In addition, long-term opioid use is associated with tolerance, dependence, and the potential for misuse, which may further worsen patient outcomes.^2)^

## Assessment and Evaluation of Chronic Postoperative Pain

The evaluation of CPTPS requires a multidimensional approach, as pain reflects not only sensory intensity but also encompasses sensory, emotional, and functional dimensions.^26)^ Initial pain assessment is commonly performed using the NRS or the Visual Analog Scale (VAS), which are simple, valid, and sensitive to changes in clinical condition. However, these instruments have limitations, as they primarily measure pain intensity without providing information on the underlying characteristics or mechanisms of pain. This complexity underscores the need for a more comprehensive approach in evaluating chronic postoperative pain.^8,12,17)^

In conditions such as CPTPS, the neuropathic component often predominates due to intercostal nerve injury. Therefore, the use of validated neuropathic pain questionnaires, such as DN4 or LANSS, is important for identifying pain characteristics, including burning sensations, tingling, and allodynia. Recent studies have shown that the DN4 is a valid and reliable screening tool with good diagnostic accuracy for identifying neuropathic pain across various clinical conditions.^27)^ Identification of this component has significant clinical implications in guiding more specific and targeted therapeutic strategies.^3,6)^

In addition, evaluating the impact of pain on quality of life and patient function is an essential component of comprehensive assessment. Instruments such as the SF-36 and EQ-5D are widely used to assess health-related quality of life, encompassing physical function, psychological well-being, and social activity. Functional assessment is also necessary to evaluate daily activities, mobility, and physical capacity, as chronic pain may lead to activity limitations, deconditioning, and reduced respiratory function.^6,4)^

In clinical practice, pain assessment should not be limited to rest but also evaluated during activities such as coughing, deep breathing, or mobilization. This dynamic pain more accurately reflects its clinical impact on respiratory function and the rehabilitation process. Therefore, a combination of assessment methods encompassing pain intensity, characteristics, and functional impact is essential to obtain a comprehensive evaluation and to support more optimal management.^9,12,21)^

## Pain Management Strategies

Management of CPTPS requires a comprehensive multimodal approach, given the complex mechanisms involving nociceptive, neuropathic, and psychological components. Pain management strategies should aim not only to reduce pain intensity but also to prevent pain chronification, improve respiratory function, and enhance long-term quality of life.^2,8,12)^

### Pharmacological management

Pharmacological therapy remains a fundamental component of perioperative pain management following thoracic surgery.^2,12)^ Simple analgesics, including paracetamol (acetaminophen) and nonsteroidal anti-inflammatory drugs (NSAIDs), are commonly used as first-line agents within multimodal analgesia protocols because of their effectiveness in reducing nociceptive pain and opioid requirements. Paracetamol may be administered orally or intravenously during the perioperative period and has demonstrated opioid-sparing effects while maintaining a relatively favorable safety profile.^2,9)^ NSAIDs also contribute to improved analgesia through inhibition of inflammatory pathways; however, their use should be individualized because of potential gastrointestinal, renal, and cardiovascular adverse effects.^2,12)^

In cases of moderate to severe pain, opioids remain an important therapeutic option, particularly during the acute postoperative phase.^2,12)^ Patient-controlled analgesia (PCA) is widely used in thoracic surgery because it allows individualized opioid administration and may improve patient satisfaction by providing greater autonomy in pain control. Nevertheless, opioid therapy and PCA are associated with important limitations, including nausea, sedation, respiratory depression, and the risk of prolonged opioid use. Therefore, opioid-sparing multimodal analgesia strategies are increasingly recommended to optimize pain control while minimizing opioid-related adverse effects.^2,8,17)^

In patients with chronic pain or predominant neuropathic components, agents such as gabapentinoids (gabapentin and pregabalin) and antidepressants, including tricyclic antidepressants and serotonin–norepinephrine reuptake inhibitors, may provide additional benefit through modulation of pain transmission pathways.^8,12,17)^ Topical analgesics may also be considered in selected patients to reduce systemic adverse effects.^12)^

Multimodal analgesia is based on the combined use of agents with different mechanisms of action to achieve synergistic pain control while minimizing adverse effects. Nevertheless, pharmacological therapy alone is often insufficient, particularly in complex or predominantly neuropathic chronic pain, thereby necessitating additional and more targeted approaches.^2,8,12)^

### Regional anesthesia and nerve block

Regional anesthesia is a key component of pain management in thoracic surgery, serving both perioperative and preventive roles in reducing pain chronification. TEA has traditionally been regarded as the gold standard for postoperative pain control because of its efficacy in blocking pain transmission via segmental neural pathways. Nevertheless, its use is associated with potential limitations, including hypotension, urinary retention, and rare but significant neurological complications.^2,9)^

PVB has emerged as an increasingly popular alternative, providing unilateral analgesia with a lower incidence of systemic side effects. Furthermore, newer regional techniques, including SAPB and ESPB, offer technically simpler and relatively safe options with favorable efficacy in managing chest wall pain. ICNBs remain an option; however, their analgesic duration is comparatively limited.^9,28)^

In addition to conventional regional anesthesia techniques, intercostal nerve cryoablation (INCA) has emerged as an adjunct modality in pain management for patients with rib fractures. This technique works by inducing temporary nerve injury through a freezing process (axonotmesis), thereby inhibiting pain transmission without damaging the supporting nerve structures. The resulting analgesic effect may last longer than that of conventional nerve blocks, as nerve regeneration occurs gradually during the healing process.^28,29)^ Evidence from several studies suggests that INCA is associated with decreased opioid consumption, enhanced pain control, and a potential reduction in hospital length of stay.^29,30)^ However, the current body of evidence remains limited, with most data derived from observational studies with relatively small sample sizes. In addition, the long-term effectiveness and the risk of complications, such as neuroma formation, are not yet fully understood. Therefore, further research is required to clarify the role of INCA in the management of CPTPS.^28,30)^ Given the complexity of chronic post-thoracic surgical pain, a multimodal approach to pain management is essential. The main pain management strategies in thoracic surgery, including their mechanisms, indications, advantages, and limitations, are summarized in **Table 3**.

**Table 3:** Pain management strategies in thoracic surgery

Modality	Mechanism	Typical indication	Advantage	Limitations
Paracetamol (acetaminophen)	Central analgesic effect	Mild-to-moderate postoperative pain; multimodal analgesia	Opioid-sparing effect; favorable safety profile	Limited efficacy as monotherapy for severe pain
NSAIDs	Inhibition of cyclooxygenase and inflammatory pathways	Mild-to-moderate nociceptive pain	Anti-inflammatory effect; opioid-sparing	Gastrointestinal, renal, and cardiovascular adverse effects
Opioids	Central opioid receptor activation	Moderate-to-severe postoperative pain	Strong analgesic effect	Sedation, respiratory depression, nausea, dependence risk
PCA	Patient-administered opioid delivery	Acute postoperative pain management	Individualized analgesia; improved patient autonomy	Opioid-related adverse effects; monitoring required
TEA	Segmental neuraxial blockade	Open thoracotomy	Gold standard analgesia; improved respiratory function	Hypotension, urinary retention, neurological complications
PVB	Unilateral thoracic nerve blockade	Thoracotomy and minimally invasive surgery	Effective analgesia with fewer systemic effects	Technical expertise required
ESPB	Fascial plane block	VATS/RATS	Technically simpler; relatively safe	Limited long-term evidence
SAPB	Lateral thoracic wall nerve blockade	Minimally invasive thoracic surgery	Reduced chest wall pain; opioid-sparing	Variable analgesic duration
ICNB	Direct intercostal nerve infiltration	VATS and localized thoracic pain	Technically simple; useful intraoperatively	Short duration of analgesia
INCA	Temporary cryogenic nerve interruption	Rib fracture and selected thoracic procedures	Prolonged analgesic effect	Limited evidence; potential neuroma formation

NSAIDs, nonsteroidal anti-inflammatory drugs; PCA, patient-controlled analgesia; TEA, thoracic epidural analgesia; PVB, paravertebral block; ESPB, erector spinae plane block; SAPB, serratus anterior plane block; ICNB, Intercostal nerve block; INCA, intercostal nerve cryoablation

### Cell-free therapy

In recent years, cell-free therapy has emerged as a promising regenerative approach for the management of chronic pain and tissue injury.^10,11)^ Unlike cell-based therapies, cell-free strategies utilize bioactive molecules and extracellular components without direct cellular transplantation.^10)^ These therapies include platelet-rich plasma (PRP), exosomes, extracellular vesicles, and various growth factors, all of which may contribute to modulation of inflammation, tissue repair, and neural regeneration.^10,11)^

PRP is an autologous blood-derived product prepared through centrifugation techniques to obtain a concentrated platelet fraction containing multiple growth factors, including platelet-derived growth factor, transforming growth factor-β, and vascular endothelial growth factor.^10)^ These bioactive mediators may promote tissue healing, angiogenesis, and modulation of inflammatory responses.^10,11)^ In neuropathic pain conditions, PRP has demonstrated potential anti-inflammatory and neuroregenerative effects, although evidence specific to CPTPS remains limited.^10)^

Exosomes and extracellular vesicles, particularly those derived from mesenchymal stem cells (MSCs), have gained increasing attention because of their role in intercellular communication and paracrine signaling.^10)^ These vesicles contain proteins, lipids, cytokines, and microRNAs that may suppress neuroinflammation, reduce peripheral sensitization, and support neural repair processes. Experimental studies suggest that exosome-based therapies may attenuate neuropathic pain mechanisms and modulate central sensitization pathways.^8,10)^

Despite these promising findings, the current clinical evidence supporting cell-free therapies in thoracic surgery remains limited.^10,11)^ Most available studies are preclinical or derived from neuropathic pain models outside the thoracic surgical setting.^10)^ In addition, challenges including variability in preparation protocols, dosing standardization, regulatory considerations, and long-term safety remain unresolved. Therefore, cell-free therapies should currently be considered investigational and require further prospective clinical studies before routine clinical implementation in patients with CPTPS.^10,11)^

### Cell-based therapy

Cell-based therapies, particularly those involving MSCs, have emerged as potential regenerative strategies for the management of chronic pain and tissue injury.^10,11)^ MSCs may be derived from various sources, including bone marrow, adipose tissue, and umbilical cord tissue, and possess immunomodulatory, anti-inflammatory, and neuroregenerative properties.^10)^ These cells exert their therapeutic effects primarily through paracrine signaling and the secretion of bioactive mediators that influence inflammatory and neural repair pathways.^10,11,13)^

Experimental studies suggest that MSCs may attenuate chronic pain through modulation of neuroinflammation, reduction of peripheral and central sensitization, and promotion of tissue regeneration.^8,10)^ In addition, MSCs may support neural repair by secreting cytokines, growth factors, and extracellular vesicles that contribute to restoration of the neural microenvironment. Various administration routes have been explored in preclinical studies, including systemic intravenous injection, local perineural administration, and intrathecal delivery, depending on the targeted pain mechanism and tissue involvement.^10)^

Despite these promising regenerative properties, the clinical application of MSC-based therapies in thoracic surgery and CPTPS remains highly limited.^10,11)^ Most currently available evidence is derived from experimental studies or chronic neuropathic pain models outside thoracic surgery.^10)^ Furthermore, several translational challenges remain unresolved, including optimal cell source selection, dosing standardization, administration protocols, long-term safety, cost, and regulatory considerations. Consequently, MSC-based therapies should currently be regarded as investigational approaches that require further prospective clinical trials before routine implementation in thoracic surgical practice.^10,11)^

### Regenerative rehabilitation

Regenerative rehabilitation is an emerging interdisciplinary approach that integrates regenerative medicine strategies with rehabilitation interventions to optimize tissue healing, functional recovery, and pain modulation. This concept emphasizes that biological repair processes and mechanical or functional stimulation may act synergistically to enhance postoperative recovery.^11)^ In the context of CPTPS, regenerative rehabilitation may provide a more comprehensive approach by addressing both persistent pain mechanisms and associated functional impairment.^2,8,11)^

Rehabilitation interventions, including respiratory exercises, early mobilization, neuromuscular retraining, and desensitization therapy, remain essential components of postoperative thoracic care. These strategies may improve pulmonary ventilation, restore chest wall mechanics, prevent physical deconditioning, and reduce maladaptive movement patterns associated with chronic pain.^11,16)^ Furthermore, gradual functional stimulation may contribute to modulation of central sensitization and improvement of pain-related disability.^11,19,20)^

From a regenerative perspective, rehabilitation may also influence biological healing processes through improvement of tissue perfusion, modulation of inflammatory responses, and stimulation of endogenous growth factor release.^11)^ When combined with biological therapies such as PRP, exosome-based therapies, or MSC-based interventions, rehabilitation may potentially enhance tissue regeneration and neural recovery through complementary mechanobiological mechanisms.^10,11)^

Despite its promising translational potential, clinical evidence supporting regenerative rehabilitation specifically in thoracic surgery remains limited.^10,11)^ Current evidence is largely extrapolated from musculoskeletal rehabilitation and experimental regenerative medicine studies.^11)^ In addition, heterogeneity in rehabilitation protocols, variability in biologic therapies, and the absence of standardized outcome measures continue to limit broad clinical implementation. Therefore, further prospective and multidisciplinary studies are required to establish the role of regenerative rehabilitation in preventing and managing CPTPS.^10,11)^ A summary of emerging therapies, including cell-based, cell-free, and regenerative rehabilitation approaches, is provided in **Table 4**.

**Table 4:** Emerging therapies for chronic pain

Therapy	Proposed mechanism	Potential benefit	Current evidence
PRP	Growth factor-mediated anti-inflammatory and regenerative effects	Tissue healing and neural recovery	Limited clinical evidence
Exosomes	Paracrine signaling and neuroinflammatory modulation	Neural regeneration and pain modulation	Experimental/preclinical
MSCs	Immunomodulatory and neuroregenerative effects	Tissue repair and reduction of sensitization	Promising but investigational
Regenerative rehabilitation	Integration of rehabilitation and regenerative medicine	Functional recovery and pain modulation	Emerging translational evidence

PRP, platelet-rich plasma; MSCs, mesenchymal stem cells

## Proposed Clinical Approach to Managing Chronic Pain after Thoracic Surgery

**Fig. 2:**
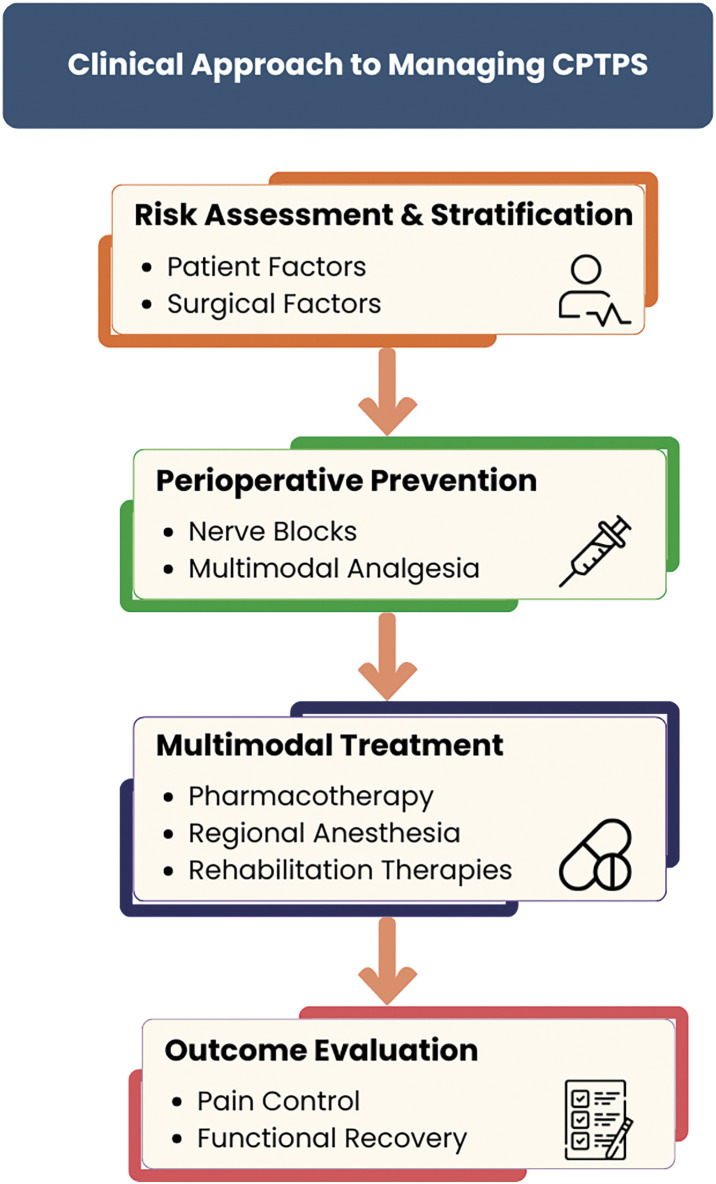
Stepwise clinical approach to the management of CPTPS, including risk assessment, perioperative prevention, multimodal treatment, and outcome evaluation. CPTPS, chronic post-thoracotomy pain syndrome

The clinical management of CPTPS should follow a structured and stepwise approach, considering risk factors, preventive measures, and stage-specific interventions. Such an approach is intended not only to alleviate pain but also to prevent chronicity and enhance functional recovery and overall quality of life.^2,8,12)^ A structured clinical approach to the management of CPTPS is illustrated in **Fig. 2**.

The first step involves identifying patients at high risk of developing chronic pain. Key factors to consider include the type of surgical procedure, particularly open thoracotomy, the presence of severe acute postoperative pain, and a prior history of chronic pain.^3,13)^ Additional factors, including psychological conditions such as anxiety and depression, may further increase the risk of pain chronification. Early recognition of these factors enables the application of targeted preventive strategies beginning in the early stages of patient care.^12,13,18)^

The second step focuses on optimizing perioperative prevention. This approach includes the use of surgical techniques that minimize tissue trauma and nerve injury, such as muscle-sparing and nerve-sparing techniques.^3,16)^ In addition, the implementation of multimodal analgesia and the use of regional anesthesia techniques, such as TEA, PVB, or other nerve blocks, play an important role in controlling acute pain.^2,9)^ This approach also contributes to the prevention of central sensitization, which plays a key role in the development of chronic pain.^8,19)^

The third step involves the management of established chronic pain. This approach should be multimodal, including pharmacological therapies such as conventional analgesics, gabapentinoids, and antidepressants for neuropathic components.^2,12,17)^ Interventional pain management strategies, including nerve blocks and other procedural techniques, may be considered in cases that are refractory to conventional treatment.^9)^ In addition, physical rehabilitation, including physiotherapy, breathing exercises, and early mobilization, is an essential component of functional recovery.^16)^ On the other hand, psychological aspects should also be addressed through psychological support and patient education, which can improve coping mechanisms and enhance adherence to therapy. This approach emphasizes that chronic pain management should be holistic, focusing not only on pain reduction but also on functional recovery and improvement of patients’ quality of life.^18)^ In selected cases, biological therapies, including cell-free and cell-based approaches, may be considered as adjunctive treatment options; however, their use remains limited and requires careful clinical consideration.^10,11)^

The fourth step involves comprehensive outcome evaluation. Assessment should not be limited to pain intensity using scales such as the NRS or VAS, but should also include respiratory function, physical activity capacity, and overall quality of life.^2,7)^ In addition, patient satisfaction with treatment outcomes is an important indicator in evaluating overall management success. Continuous evaluation allows for dynamic adjustment of therapy based on the patient’s clinical response.^1,12)^

Overall, this stepwise clinical approach highlights the importance of integrating prevention, treatment, and outcome evaluation in the management of CPTPS. Through a systematic and multidimensional strategy, it is expected that the incidence of CPTPS can be reduced and patients’ quality of life can be optimally improved.^3,8)^

Integration of chronic pain prevention strategies into enhanced recovery after surgery (ERAS) pathways may further optimize postoperative outcomes following thoracic surgery.^16)^ Contemporary thoracic ERAS protocols emphasize multimodal analgesia, opioid-sparing strategies, minimally invasive surgical approaches, early mobilization, respiratory rehabilitation, and standardized perioperative care.^2,16)^ These interventions may reduce acute postoperative pain, attenuate central sensitization, and potentially decrease the transition from acute postoperative pain to CPTPS.^8,16)^ In addition, regional anesthesia techniques such as TEA, PVB, and newer fascial plane blocks may contribute to improved respiratory function and enhanced postoperative recovery.^2,9)^ Therefore, integration of chronic pain prevention principles into ERAS pathways may represent an important strategy for improving long-term patient outcomes and quality of life after thoracic surgery.^16)^

## Impact of Adequate Pain Intervention on Patient Satisfaction and Quality of Life

Adequate pain management after thoracic surgery plays a central role in shaping the overall patient experience. Uncontrolled pain can reduce comfort, increase anxiety, and negatively affect patients’ perception of surgical success. Conversely, optimal pain control contributes to a more positive care experience and enhances patient satisfaction with treatment outcomes.^2)^ In this context, pain management serves not only as a symptomatic intervention but also as a key component of patient-centered care.

Furthermore, pain is closely associated with the process of functional recovery. Adequately controlled pain enables patients to perform deep breathing, effective coughing, and early mobilization, all of which are essential in preventing pulmonary complications and accelerating respiratory recovery. Conversely, persistent pain may lead to respiratory inhibition, reduced activity, and deconditioning, ultimately delaying rehabilitation and increasing the risk of postoperative complications.^16)^

The impact of pain also extends to daily life, including sleep quality, mobility, and the ability to perform routine activities. Chronic pain can disrupt sleep patterns, reduce energy levels, and limit social interaction and productivity. Over the long term, these effects contribute to a decline in overall quality of life.^23,24)^ Therefore, outcome evaluation based on patient-reported outcomes (PROs), such as quality of life and daily functioning, is essential for comprehensively assessing the effectiveness of pain management.

Overall, adequate pain intervention not only reduces pain intensity but also improves quality of life and patient satisfaction with surgical outcomes. An effective and multidimensional approach to pain management enables more optimal recovery, encompassing both physiological and psychosocial aspects, thereby providing meaningful long-term benefits for patients after thoracic surgery.

## Current Challenges and Future Perspectives

Despite advances in the understanding of CPTPS, several challenges remain in both clinical practice and research. One of the primary challenges is the heterogeneity in the definition of chronic postsurgical pain, particularly regarding pain duration and diagnostic criteria, which leads to variability in reported prevalence and complicates comparisons across studies.^12)^ In addition, the lack of standardized analgesic protocols, both in terms of intervention types and treatment duration, contributes to variability in reported outcomes across the literature.

Another significant challenge is the limited scientific evidence supporting newer therapeutic approaches, such as cell-free and cell-based therapies. Although early studies demonstrate potential in modulating inflammation and promoting tissue regeneration, most of the available evidence is derived from experimental or observational studies with relatively small sample sizes.^10)^ Specifically, studies evaluating the effectiveness of these therapies in patients undergoing thoracic surgery remain very limited, warranting caution in their clinical application.

Furthermore, most studies on chronic postsurgical pain are heterogeneous and often not specific to thoracic surgery. Much of the available data is derived from mixed surgical populations, thereby limiting its generalizability to the context of thoracic surgery.^3)^ Therefore, well-designed prospective studies, including multicenter studies and randomized controlled trials, are needed to generate more consistent and clinically applicable evidence.

Future directions in the management of chronic pain after thoracic surgery are expected to increasingly emphasize personalized and mechanism-based approaches.^8,12,13)^ Advances in perioperative risk stratification, pain phenotyping, and biomarker research may help identify patients at higher risk of developing CPTPS, thereby enabling earlier and more targeted preventive interventions.^8)^ In addition, the integration of multimodal analgesia, minimally invasive surgical techniques, rehabilitation, and emerging regenerative therapies may support the development of more individualized treatment strategies.^2,16)^

Translational research also remains essential to bridge the gap between experimental findings and clinical implementation. Although regenerative approaches such as PRP, exosome-based therapies, and mesenchymal stem cell interventions demonstrate promising anti-inflammatory and neuroregenerative potential, their long-term efficacy, safety, and feasibility in thoracic surgical populations remain uncertain.^10,11)^ Future prospective multicenter studies and randomized controlled trials are needed to establish standardized protocols, optimize patient selection, and clarify the role of these therapies in routine clinical practice.

Ultimately, multidisciplinary collaboration among thoracic surgeons, anesthesiologists, pain specialists, rehabilitation physicians, and researchers will be crucial for improving long-term postoperative outcomes and quality of life in patients undergoing thoracic surgery.

## Conclusion

CPTPS remains a significant complication after thoracic surgery that adversely affects respiratory function, physical activity, and quality of life. Its development is multifactorial, involving tissue injury, intercostal nerve damage, inflammatory responses, and peripheral and central sensitization. Therefore, effective prevention and management require a comprehensive multidisciplinary approach integrating optimized surgical techniques, multimodal analgesia, regional anesthesia, rehabilitation, and individualized perioperative care. Advances in minimally invasive surgery, opioid-sparing strategies, and ERAS protocols have improved postoperative pain management and functional recovery, while emerging regenerative approaches, including cell-free therapy, cell-based therapy, and regenerative rehabilitation, demonstrate promising translational potential despite still limited clinical evidence in thoracic surgery. Future efforts should focus on personalized pain management, improved perioperative risk stratification, and multidisciplinary collaboration to optimize long-term outcomes and quality of life after thoracic surgery.

## Declarations

### Data availability

Data sharing is not applicable to this article as no datasets were generated or analyzed during the current study.
